# Supplementary material for: Gut Microbiota Mediates Skin Ulceration Syndrome Outbreak by Readjusting Lipid Metabolism in Apostichopus japonicus
Source: Int J Mol Sci. 2022 Nov 5;23(21):13583. doi: 10.3390/ijms232113583 (PMC9653642; doi:10.3390/ijms232113583)
Supplement: Supplementary file 1 [file ijms-23-13583-s001.zip › ijms-1954800-supplementary.pdf]

Supporting Materials

Table S1. Primers used in this study.

Table S2. Analysis of similarity (ANOSIM) test the dissimilarities of sea cucumber gut microbiotas using Bray-Curit distance.

Table S3. Bacterial taxonomic profiling at the phylum level. Data are represented as the mean ± SD (n=8).

Table S4. Bacterial taxonomic profiling at the family level. Data are represented as the mean ± SD (n=8).

Table S5. The top 25 variables were extracted in positive and negative ion modes by OPLS-DA analysis, respectively.

Figure S1. OPLS loading S-plot for the total lipids of the 12 h, 48 h, 96 h and SUS groups and the negative control group detected in the (A) ESI^+ and (B) ESI^- modes.

Table S1. Primers used in this study.

Biomarker	Primers sequences-upstream	Primers sequences-downstream
Aj β-actin	CCATTCAACCCTAAAGCCAACA	ACACACCGTCTCCTGAGTCCAT
Aj IL17	GTTTGTGGTGCTGTTCTCTGTGA	GGACTTCGATCGGGTCTTTTG
Aj p105	TCTTCGCATTCCATTGAGCTG	ATGGTCCTTCACAGCCGTATCT
Aj TGFβ	GGGATCTCGATTGGAAGTGG	GGTGAGGCGTTAGGGTTTGT
Aj VEGF	AGCCTAGGCCTGTGCTCCTA	CCACTTGCGCTGTATATACTTTCA
Aj MMIF	ATTTCAGAGTTTGCGGAGGA	TATTCGGGCTCAGCTTGAGACC

Table S2. Analysis of similarity (ANOSIM) test the dissimilarities of sea cucumber gut microbiotas using Bray-Curit distance.

	Control		12 h		48 h		96 h		SUS	
	r	*P*	r	*P*	r	*P*	r	*P*	r	*P*
Control	—	—								
12 h	0.5502	**0.002**	—	—						
48 h	0.8415	**0.0002**	0.8119	**0.0002**	—	—				
96 h	0.8426	**0.0001**	0.9911	**0.0005**	0.856	**0.0004**	—	—		
SUS	0.8421	**0.0004**	0.793	**0.0004**	0.4883	**0.0006**	0.1345	0.0521	—	—

Table S3. Bacterial taxonomic profiling at the phylum level. Data are represented as the mean ± SD (n=8).

Phylum	Control	12 h	48 h	96 h	SUS
Proteobacteria	61.55±8.53	49.03±6.39	45.90±5.44	71.39±4.56	74.80±14.79
Firmicutes	14.57±10.79	33.35±8.75	38.21±6.42	8.74±1.31	12.52±12.97
Bacteroidetes	8.46±3.61	6.13±2.24	9.41±3.77	9.82±5.01	7.49±5.01

Actinobacteria	9.57±1.50	8.66±2.38	3.52±0.98	3.49±1.07	2.26±1.65
Verrucomicrobia	3.08±1.80	1.12±0.57	1.70±0.77	3.81±2.14	1.63±0.95
Fusobacteria	0.10±0.09	0.14±0.08	0.48±0.40	2.11±4.25	0.93±0.80
Chloroflexi	0.82±0.40	0.48±0.16	0.22±0.17	0.19±0.09	0.13±0.10
Acidobacteria	0.34±0.08	0.29±0.10	0.15±0.05	0.14±0.06	0.06±0.06
Others	1.50±0.16	0.80±0.22	0.41±0.15	0.30±0.12	0.19±0.19

Table S4. Bacterial taxonomic profiling at the family level. Data are represented as the mean ± SD (n=8).

Family	Control	12 h	48 h	96 h	SUS
Rhodobacteraceae	19.76±6.52	11.51±3.47	20.89±4.81	47.13±11.65	47.81±21.88
Flavobacteriaceae	6.65±3.64	4.00±2.01	8.84±3.84	8.93±5.04	6.65±5.56
Desulfobulbaceae	5.17±3.65	2.31±2.33	1.45±0.44	0.55±0.16	0.53±0.52
Vibrionaceae	2.19±1.16	4.76±2.16	5.49±2.78	4.89±2.48	5.40±8.43
Bacillaceae	3.83±3.10	21.48±8.76	34.40±6.78	4.56±1.09	9.21±12.11
Pseudoalteromonadaceae	3.30±2.71	5.81±2.01	1.46±0.80	0.97±0.42	0.84±0.69
Verrucomicrobiaceae	3.05±1.81	1.08±0.59	1.68±0.77	3.80±2.14	1.63±0.95
Planococcaceae	2.69±1.94	4.62±1.20	1.44±0.85	1.66±0.64	0.95±0.73
OM60	7.22±4.94	1.58±0.63	1.36±0.34	0.88±0.24	0.82±0.79
Psychromonadaceae	0.71±0.49	0.88±0.47	5.34±2.72	5.18±6.24	7.11±4.20
Phyllobacteriaceae	0.53±0.13	1.40±1.30	1.54±1.52	4.32±3.87	5.91±8.11
Others	44.89±12.31	40.58±7.53	16.11±3.46	17.13±4.77	13.14±6.89

Table S5. The top 25 variables were extracted in positive and negative ion modes by OPLS-DA analysis, respectively.

**m/z**	**BaseRt**	**Class**	**FA**	**Formula**	**MainIon**	**VIP**
**ESI$^+$**						
903.7404	27.041	TG	18:1/18:2/18:2	C57 H100 O6	+Na	6.55
901.7253	25.072	TG	18:2/18:2/18:2	C57 H98 O6	+Na	6.52
850.7409	29.432	TG	16:0/16:0/18:1	C53 H100 O6	+NH4	5.98
877.7256	26.865	TG	16:0/18:2/18:2	C55 H98 O6	+Na	5.46
905.7569	29.336	TG	18:0/16:0/20:4	C57 H102 O6	+Na	4.76
905.7567	28.966	TG	18:1/18:1/18:2	C57 H102 O6	+Na	4.59
879.7409	28.883	TG	16:0/18:1/18:2	C55 H100 O6	+Na	4.17
878.8166	29.752	TG	18:0/16:0/18:1	C55 H104 O6	+NH4	3.98
639.4954	11.996	DG	18:2/18:2	C39 H68 O5	+Na	3.39
822.7564	28.633	TG	16:0/14:0/18:1	C51 H96 O6	+NH4	3.33
824.7701	29.399	TG	16:0/16:0/16:0	C51 H98 O6	+NH4	3.31
823.6786	24.281	TG	16:1/16:1/16:1	C51 H92 O6	+Na	3.18
825.6937	26.287	TG	16:0/16:1/16:1	C51 H94 O6	+Na	3.02
903.7435	27.598	TG	18:1/18:1/18:3	C57 H100 O6	+Na	2.89
566.551	13.865	Cer	d22:1/14:0	C36 H71 O3 N1	+H	2.64
801.6943	28.569	TG	16:0/14:0/16:0	C49 H94 O6	+Na	2.58
852.8012	29.745	TG	18:0/16:0/16:0	C53 H102 O6	+NH4	2.50

875.7101	24.913	TG	16:1/18:2/18:2	C55 H96 O6	+Na	2.36
799.678	26.165	TG	16:0/14:0/16:1	C49 H92 O6	+Na	2.25
899.7092	23.568	TG	18:3/18:2/18:2	C57 H96 O6	+Na	2.22
906.8484	29.944	TG	20:0-16:0-18:1	C57 H108 O6	+NH4	2.17
568.5662	15.544	Cer	d22:0/14:0	C36 H73 O3 N1	+H	2.09
877.7254	27.448	TG	16:0-16:1-20:3	C55 H98 O6	+Na	1.91
871.6784	22.64	TG	16:1/16:1/20:5	C55 H92 O6	+Na	1.83
864.8014	29.592	TG	16:0/17:0/18:1	C54 H102 O6	+NH4	1.81
**ESI$^-$**						
555.2849	1.67	SQMG	16:0	C25 H48 O11 S1	-H	7.51
688.4931	10.198	PE	16:0/16:1	C37 H72 O8 N1 P1	-H	3.46
719.488	11.396	PG	16:0/16:1	C38 H73 O10 N0 P1	-H	3.35
745.5036	11.876	PG	16:0/18:2	C40 H75 O10 N0 P1	-H	3.27
481.2576	1.49	LPG	16:1	C22 H43 O9 N0 P1	-H	3.08
450.2631	1.311	LPE	16:1	C21 H42 O7 N1 P1	-H	2.97
714.509	10.461	PE	16:1/18:1	C39 H74 O8 N1 P1	-H	2.67
483.2734	1.873	LPG	16:0	C22 H45 O9 N0 P1	-H	2.59
507.315	1.578	LPG	18:2	C24 H45 O9 N0 P1	-H	2.43
686.4776	7.996	PE	16:1/16:1	C37 H70 O8 N1 P1	-H	2.31
452.2788	1.622	LPE	16:0	C21 H44 O7 N1 P1	-H	2.24
717.4722	9.062	PG	16:1/16:1	C38 H71 O10 N0 P1	-H	2.18
563.3443	1.78	MGMG	18:1	C27 H50 O9	+HCOO	2.17
714.5087	11.075	PE	16:0/18:2	C39 H74 O8 N1 P1	-H	2.08
509.2889	1.957	LPG	18:1	C24 H47 O9 N0 P1	-H	1.98
476.2786	1.37	LPE	18:2	C23 H44 O7 N1 P1	-H	1.97
838.5974	12.007	PC	18:1/20:4	C46 H84 O7 N1 P1	+HCOO	1.86
478.2941	1.675	LPE	18:1	C23 H46 O7 N1 P1	-H	1.80
740.5244	10.875	PE	18:1/18:2	C41 H76 O8 N1 P1	-H	1.72
850.5611	8.554	PC	18:1/20:5	C46 H80 O8 N1 P1	+HCOO	1.66
581.3002	1.748	SQMG	18:1	C27 H50 O11 S1	-H	1.64
743.4877	9.515	PG	16:1/18:2	C40 H73 O10 N0 P1	-H	1.61
747.5191	14.253	PG	16:0/18:1	C40 H77 O10 N0 P1	-H	1.52
716.5247	12.879	PE	16:0/18:1	C39 H76 O8 N1 P1	-H	1.47
771.519	12.144	PG	18:1/18:2	C42 H77 O10 N0 P1	-H	1.46

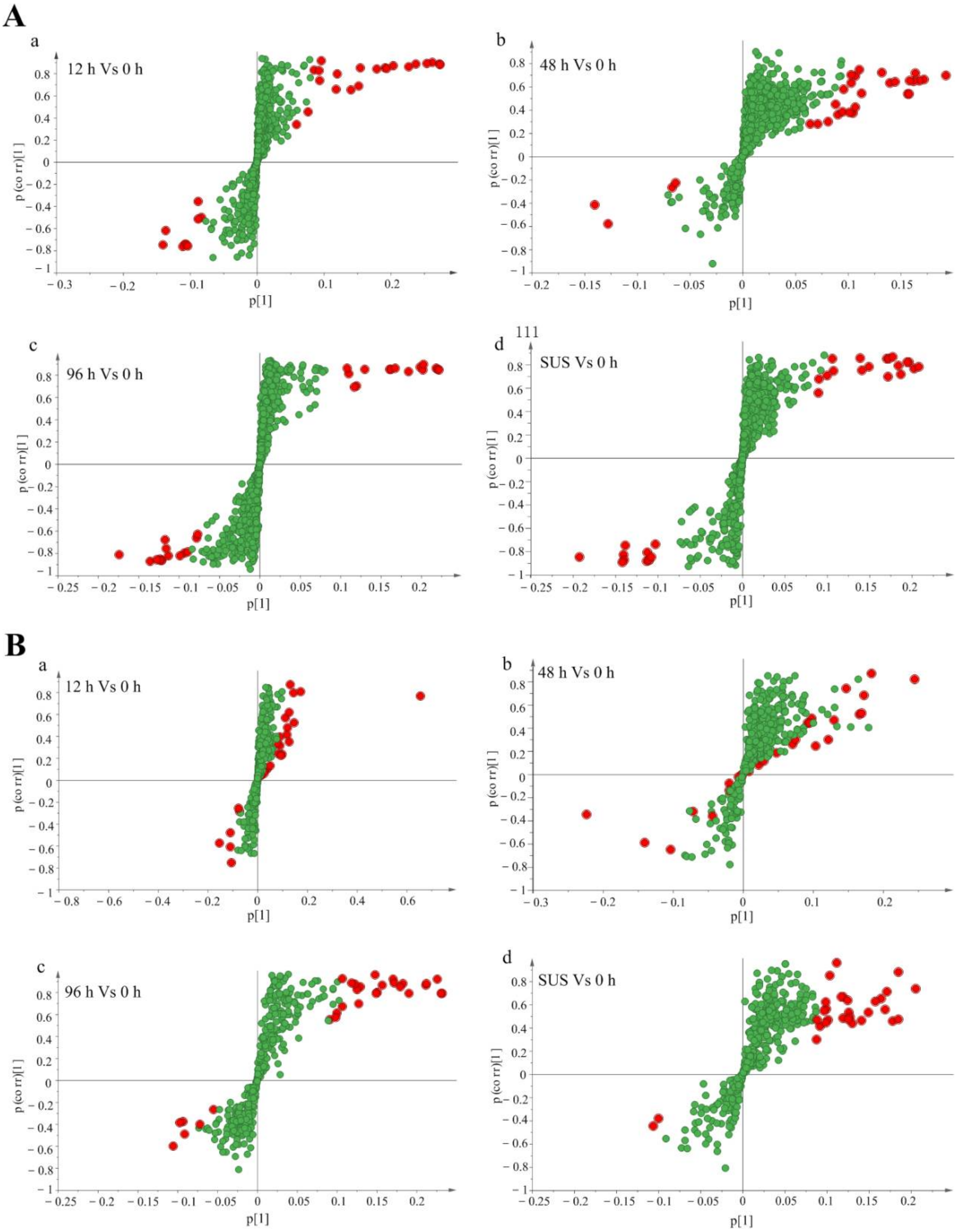

Figure S1. OPLS loading S-plot for the total lipids of the 12 h, 48 h, 96 h and SUS groups and the negative control group detected in the (**A**) ESI$^+$ and (**B**) ESI$^-$ modes.
